# The Bright and Dark Side of DNA Methylation: A Matter of Balance

**DOI:** 10.3390/cells8101243

**Published:** 2019-10-12

**Authors:** Marta Borchiellini, Simone Ummarino, Annalisa Di Ruscio

**Affiliations:** 1 Department of Health Sciences, University of Eastern Piedmont, 28100 Novara, Italy; marta.borchiellini@uniupo.it; 2 Department of Translational Medicine, University of Eastern Piedmont, 28100 Novara, Italy; 3 Harvard Medical School Initiative for RNA Medicine, Harvard Medical School, Boston, MA 02115, USA

**Keywords:** epigenetics, DNA methylation, DNMTs, imprinting, cancer

## Abstract

DNA methylation controls several cellular processes, from early development to old age, including biological responses to endogenous or exogenous stimuli contributing to disease transition. As a result, minimal DNA methylation changes during developmental stages drive severe phenotypes, as observed in germ-line imprinting disorders, while genome-wide alterations occurring in somatic cells are linked to cancer onset and progression. By summarizing the molecular events governing DNA methylation, we focus on the methods that have facilitated mapping and understanding of this epigenetic mark in healthy conditions and diseases. Overall, we review the bright (health-related) and dark (disease-related) side of DNA methylation changes, outlining how bulk and single-cell genomic analyses are moving toward the identification of new molecular targets and driving the development of more specific and less toxic demethylating agents.

## 1. DNA Methylation: More than One Purpose

**Figure 1:**
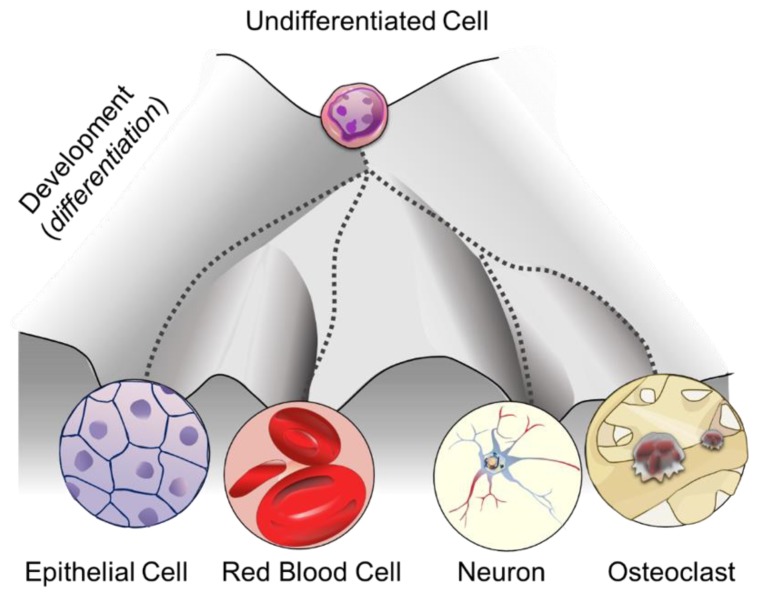
An outline depicting cell-fate plasticity according to the Waddington’s epigenetic landscape (inspired by the model proposed by Waddington [3]).

DNA methylation is a key epigenetic signature (Box 1, Figure 1) implicated in regulation of gene expression that occurs predominantly within CpG dinucleotides [1,2,3,4,5,6]. CpG dinucleotides are under-represented in the mammalian genome (1%), but tend to cluster in CpG-rich regions called CpG islands (CGIs), located in the proximity of the transcription start sites (TSSs) of the majority (70%) of human protein-coding genes [7,8]. CGIs are stretches of DNA sequences of 200 nucleotides or greater [9], with the GC ratio observed/expected to be greater than 0.6. Although the bulk of genome is methylated at 70–80% of its CpGs, CGIs are mostly unmethylated in somatic cells [9,10].

**Figure 2:**
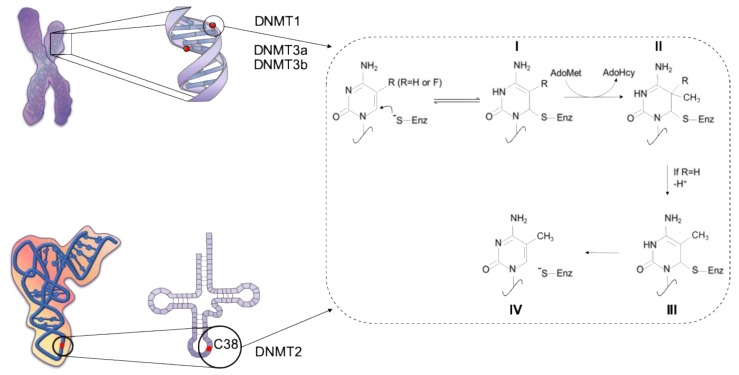
Schematic representation of the methylation reaction catalyzed by the DNA methyltransferases (DNMTs) (adapted from [14]). Shown are the mechanisms proposed for methylation of cytosine by DNMT1, 3a and 3b on DNA (upper left panel) or by DNMT2 on RNA (lower left panel). Briefly, a thiol group (SH) from the binding site of the enzyme provides the nucleophilic attack to position 6 of the cytosine heterocycle, to activate position 5 towards one-carbon transfer (I). The methyl group on position 5 is donated by the coenzyme AdoMet (II). A proton in position 5 of the 5,6-dihydropyrimidine is then removed (II–III), and a consequent β-elimination generate 5-methylcitosyne and free enzyme (IV).

DNA methylation is mediated by members of the DNA methyltransferase (DNMT) family that can covalently transfer a methyl group (CH_3_) from the universal donor S-adenosyl-L-methionine (SAM) to the carbon 5-position of the cytosine ring. Conventionally, DNMTs are classified as de novo (DNMT3a and DNMT3b) or maintenance (DNMT1) enzymes [11]. Of note, DNMT2, a member of the methyltransferase family, catalyzes methylation of RNA at position 38 in tRNAAsp GUC [12,13,14] (Figure 2). DNMT3-like (DNMT3L) is a DNMT3-associated protein lacking an enzymatic domain and interacting with DNMT3a/3b to modulate its activity [15,16]. In mice, Dnmt1 and Dnmt3b are essential for embryonic development, as DNA methylation changes dramatically. An overall demethylation after fertilization is followed by de novo methylation of discrete regions upon implantation [17]. *Dnmt1* knockout mice show early lethality at embryonic day (E) 9.5, whereas Dnmt3b depletion induces death at E 14.5–18.5, due to developmental impairment. On the contrary, Dnmt3a knockout mice do not display defects in embryonic development, but they do die at 4 weeks of age. Although this binary classification is convenient, the function of the de novo and maintenance DNMTs overlaps in many instances [18,19,20].

Box 1 **History of epigenetics.**

The term epigenetics was originally coined in the 17th century by the physician and physiologist William Harvey to indicate the gradual development of the embryo from a homogeneous to a heterogeneous material, referred to as “epigenesis” [1]. Later on, in the 1940s, Conrad Waddington used the term “epigenetics” to explain the relationship between the genotype, defined as the whole genetic system of an organism, and the phenotype, indicating the entire set of characteristics that an organism develops over time [2]. Waddington established the first causal relationship between genes and their outcomes by introducing the concept of the “epigenetic landscape” as “the various developmental pathways that undifferentiated cells (sharing identical genotype) might take toward differentiation” (Figure 1) [3]. In other words, he described how the static information written in the form of nucleotide sequences is dynamically translated into tissues and organs, thus driving cell fate decisions [4]. In the last two decades, the definition of epigenetics has evolved from “the study of mitotically and/or meiotically heritable changes in gene function that cannot be explained by changes in DNA sequence” [5] to “the structural adaptation of chromosomal regions so as to register, signal or perpetuate altered activity states”, which is inclusive of all the stable or transient chromosomal markers arising in response to different stimuli [6].

A number of studies have shown that DNA methylation is not randomly distributed across the genome, but displays regional specificity [21]. Methyl groups promote conformational changes in the major groove of DNA, thus altering protein-DNA binding [22] and, as a result, gene expression. Most studies have initially focused on the effect of CGI methylation within the promoter and TSS of protein-coding genes. Recently [23], however, more comprehensive genome-wide methylation analyses have started elucidating the role of DNA methylation at CpG clusters within exons, introns, and intergenic sequences, expanding on the previous knowledge of CGIs and leading to the identification of CG shores (regions up to 2 kb from CGI), shelves (regions from 2 to 4 kb from CGI), and open sea (the rest of the genome) regions [24,25,26].

Hence, DNA methylation needs to be framed in the context of the genomic location. As a proof of concept, methylation of TSS-associated CGIs negatively correlates with gene expression, leading to long-term gene silencing [7], whilst gene-body methylation positively correlates to gene expression [27,28,29,30]. Another interesting finding emerging from bulk methylome studies is that non-CGI-CpGs are mostly methylated and therefore less stable than CGIs, due to the tendency of 5-methylcytosine (5mC) to undergo spontaneous or enzymatic deamination to T [31]. The C-to-T transition causes germ-line or somatic mutations, resulting in the depletion of CpGs dinucleotides in the human genome. Methylation at other genomic regions, such as enhancers and insulators, does not follow a specific pattern and may vary in different settings. Enhancers and insulators are long-range regulatory elements able to alter gene expression or protect gene promoters from inappropriate signals, respectively [32,33]. Aran et al. have shown that distal methylation sites in estrogen receptor (ER)-positive breast tumors associates with breast cancer-related gene expression better than promoter methylation [34]. Moreover, Tatetsu et al. demonstrated that aberrant methylation of the 17-kb 5′ upstream enhancer of PU.1 is required for myeloma cell growth [35]. Insulators are bifunctional instead, acting either as a blocking enhancer, by preventing enhancer-mediated transcription, or as barriers, by limiting the advance of nearby heterochromatin that would otherwise silence expression [36]. CCCTC-binding factor (CTCF), an enhancer-blocking protein, does not bind to its DNA consensus sequence if methylated, as demonstrated for the imprinted *IGF2-H19* locus and the *CD45* gene [37]. It follows that DNA methylation can regulate gene expression indirectly by controlling access of enhancers to gene promoters [38]. Finally, CGI shores, with a lower CpG density, have recently emerged as critical regulatory elements affecting gene expression depending on their DNA methylation profile [39].

This review will address the impact of cutting-edge next-generation sequencing technologies on our perception and interpretation of DNA methylation in health conditions and diseases.

## 2. DNA Methylation Analysis: Think Globally, Act Locally

DNA methylation has a crucial role in various biological processes, such as development, differentiation, and gene expression [40,41,42]. Thus, comprehensive mapping becomes critical for addressing the functional role of this modification [43]. Various strategies have been developed to differentiate methylated and non-methylated C residues [44]. The initial lack of genome-wide approaches has restricted DNA methylation profiling to gene-specific evaluation using polymerase chain reaction (PCR) amplification of the target sequence. As DNA polymerases do not discriminate between C and 5mC, all potential differences in methylation are lost during classical PCR amplification [44,45]. To overcome this challenge, Frommer et al. developed a locus-specific method based on DNA treatment with sodium-bisulfite (SB) that leads to the conversion of all unmodified cytosine to uracil [45]. 5mC are resistant to deamination induced by SB, and are preserved during the PCR amplification by primers designed on the converted DNA. The resulting product is then analyzed by Sanger sequencing. Although very laborious, bisulfite sequencing PCR (BSP) is considered the gold standard to quantitatively study gene-locus specific DNA methylation [46].

The possibility to interrogate the entire genome using next generation sequencing technologies has expanded DNA methylation analyses [47]. The application of massive parallel sequencing to bisulfite-treated DNA has resulted in new genome-scale and -wide protocols, such as reduced representation bisulfite sequencing (RRBS) and whole genome bisulfite sequencing (WGBS), respectively. RRBS uses MspI, a methylation insensitive enzyme, to produce small fragments with CpG dinucleotides at both ends. Digested products are bisulfite converted and then sequenced [48]. Even though RRBS is more capable of covering a higher number of CpG loci within a given region than array-based techniques [47,49], the coverage across corresponding CpG-rich sequences might change among the samples tested, therefore introducing higher inter-sample variability and altering reproducibility [50]. As RRBS is biased for CpG-rich regions, such as CGIs, the coverage drops for CG shores, shelves, and open sea regions [50].

WGBS couples bisulfite-conversion of genomic DNA with high-throughput sequencing [51] and therefore provides a comprehensive and quantitative analysis of DNA methylome at single nucleotide resolution, without relying on restriction enzyme enrichment. Until few years ago, the high amount of DNA required as input, as well as the cost, limited its use. The recent advancement in sequencing platforms and sample preparation have now made WGBS more accessible and feasible, especially cost-wise, to allow projects with large sample sizes [52].

Likewise, non-bisulfite-based methods have also benefited from the introduction of high-throughput sequencing platforms. For example, immunoprecipitation of methylated DNA with an antibody recognizing 5mC residues coupled with sequencing (methylated DNA immunoprecipitation sequencing [MeDIP-Seq]) [47], is commonly used as an alternative approach to RRBS (Box 2).

MeDIP-Seq covers a higher number of regions than those normally screened by RRBS, but its efficiency is based on the specificity of the antibody, which can be biased toward hypermethylated sequences. Yet, neither RRBS nor MeDIP-Seq can determine the profile of virtually all CpG dinucleotides throughout the genome. Therefore, WGBS is considered the gold standard for full methylome analysis. More recently, the establishment of enzymatic methyl-Seq (EM-Seq), has added another free-bisulfite-based method to profile the entire methylome in instances when BS treatment is not suitable (i.e., fragmented DNA) or the DNA input is too low. EM-Seq relies on two sequential enzymatic reactions protecting 5mC and 5hmC from downstream deamination. The enzymatically converted DNA can then be processed for sequencing similar to WGBS [53].

**Figure 3:**
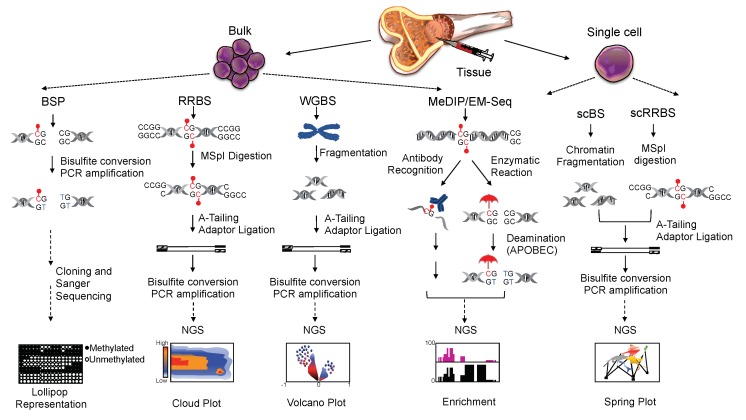
Workflows of bisulfite and non-bisulfite-based methods applied to either bulk- or single-–cell population for DNA methylation analyses. On the left hand-side, bulk-bisulfite-based methods: bisulfite sequencing PCR (BSP), reduced representation bisulfite sequencing (RRBS), and whole genome bisulfite sequencing (WGBS). Following bulk-non-bisulfite-based methods: methylated DNA immunoprecipitation sequencing (MeDIP-Seq) and enzymatic methyl sequencing (EM-Seq), in which an enzymatic reaction protects [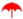] mC and/or hmC from the deamination by APOBEC. On the right hand-side, single-cell bisulfite-based methods as indicated: single-cell bisulfite sequencing (scBS) and single-cell reduced representation bisulfite sequencing (scRRBS). The red dot (●) indicates methylated cytosine.

Despite their sensitivity, all bulk sequencing methods are unable to dissect intra-cellular and intra-tumoral epigenetic heterogeneity within a specific cell population [54]. Single-cell technology has emerged as an invaluable tool to ascertain this heterogeneity [55]. WGBS and RRBS protocols have been optimized to carry out single-cell bisulfite sequencing (scBS) and single-cell RRBS (scRRBS), respectively [56]. While scBS is able to cover a higher number of CpG sites than scRRBS, the latter enables a better coverage of CGIs [57] (Figure 3).

Notably, these new approaches are shedding light on the impact of epigenetic alterations in specific cellular subsets with respect to developmental processes and cancer, filling the gaps arising from bulk studies [58].

Box 2 **More about sodium bisulfite treatment.**

Although bisulfite sequencing approaches are considered the gold standard for DNA methylation analysis, all bisulfite-based methods share common limitations. Sodium bisulfite is a harsh chemical treatment that degrades template DNA, thus producing a poor quality product. An incomplete conversion of cytosine to uracil may also introduce artifacts into the analysis [59]. In addition, bisulfite-based methods are not able to discriminate between 5-methylcitocine (5mC) and its oxidative derivative 5-hydroxymethylcytosine (5hmC), produced by the ten-eleven translocation (TET) family of dioxygenases during active DNA demethylation. Other than being a chemical intermediate, 5hmC is a stable DNA modification that binds to specific regulatory proteins and is mainly found within actively transcribed genes. 5hmC marks a decrease in cancer tissues, suggesting its potential regulatory role in the mammalian genome [60]. To overcome the limitations associated with bisulfite-based approaches, new bisulfite-free techniques [47,59] have been developed that preserve genomic DNA integrity, as well as other strategies able to distinguish 5mC and 5hmC marks [61,62].

## 3. DNA Methylation in Health: A Matter of Location and Timing

DNA methylation plays a critical role in the regulation of early development in humans and other mammals. In contrast to DNA sequences, DNA methylation is not inherited from gametes, as the parental DNA methylation pattern is erased at an early embryo stage [63]. During implantation, the DNA methylation profile is re-established, and the entire genome undergoes de novo methylation with the exclusion of CpG island-like regions, which elude this epigenetic modification due to the presence of RNA polymerase complexes that prevent access of de novo methylation machinery to the DNA. The resulting bimodal pattern is conserved throughout development and preserved for the whole lifespan of the organism unless unexpected alterations [64,65]. Yet, a group of genes, so-called “imprinted” genes, can escape this extensive reprogramming process occurring during embryonic development. Genomic imprinting is defined as the monoallelic, parental-specific expression of a gene in diploid cells, and as such, it is considered a form of gene regulation. Imprinted genes are epigenetically marked, i.e., “imprinted”, within differentially methylated regions (DMRs) in gametes, and such imprints are conserved after fertilization [66]. As a result, only one parental copy of the imprinted gene will be expressed, whereas the other copy will be silenced by DNA methylation [67,68,69]. The human insulin-like growth factor II (*IGF-II*)*/H19* region is an example of paternally imprinted gene, in which methylation of the imprinting control sequence (ICR) regulates binding of the CTCF [70,71]. In mammals, the majority of imprinted genes affects growth of the embryo, placenta, and neonate. In that regard, paternally imprinted genes function as growth enhancers, whereas maternally imprinted genes function as growth repressors [72]. The loss of maternal DNA methyltransferases results in post-implantation lethality [16,72], suggesting the essential role of genomic imprinting in embryonic development and differentiation. Another category of imprinted genes include those involved in neurologic and behavioral regulation [73].

DNA methylation also partakes in protecting the structural integrity of the genome. It has been proposed that methylation of CpGs within parasitic DNA elements and retrotransposons, which account for 40% of the entire genome, operates as a genome defense system, in order to prevent the expression of these elements and preserve genomic stability [74].

Another puzzling and intriguing matter is whether the dynamic changes observed in response to biological and non-biological stimuli are paralleled by DNA methylation changes during the different phases of the cell cycle in physiological conditions. Substantial data with respect to this question are still lacking, and the few reported studies do not seem to agree with one another. Single-cell RNA sequencing results from murine embryonic stem cells (mESCs) point to a higher methylation rate in G1/S compared to other phases of the cycle, in line with previous observations in HeLa cells and human primary foreskin fibroblasts [75,76]. Similarly, Desjorbet et al. have demonstrated that the inheritance of DNA methylation marks efficiently occurs in late S phase, and cytosine methylation is completed in G2/M phase [77]. However, a different study analyzing DNA methylation levels in G0, G1, and G2 phases in low passage primary dermal fibroblasts did not confirm these cell cycle changes in DNA methylation patterns [78]. The specific cell type interrogated, along with not including the S phase in the analysis, could possibly account for these contrasting results, since hemi-methylated DNA sequences are challenging to evaluate with low coverage sequencing. Further analyses will need to delve into this question.

## 4. DNA Methylation and Disease: Too Little, too Much, or Both?

Given its critical role in many biological processes, it is not surprising that dysregulation of DNA methylation is frequently linked to either germ-line or somatic diseases [79,80]. The study of monozygotic (MZ) twins has offered an exceptional tool to investigate the contribution of the epigenetic load to phenotypic variations, including predisposition to pathological conditions, in individuals with identical genetic background [81,82,83,84]. MZ twin studies have shown in many instances that phenotypic variations can be ascribed to differences in DNA methylation [84]. Following up, studies from the recent NASA research on MZ twins demonstrated that global DNA methylation was altered in response to different environmental stimuli, namely the absence of gravity for the one subject who was sent into space, versus the gravity the sibling who remained on earth was exposed to, affecting specific pathways transiently or permanently [85] (Box 3).

Box 3 **Environmental factors “another variation on the theme”.**

Major clues have been brought about by the evidence that the environment and environmental factors can profoundly affect DNA methylation. To list few examples, prenatal or chronic exposure to chemicals, including air pollution and tobacco smoke, or drugs associated with either global or site-specific DNA methylation alterations [86,87]. Nutritional elements, also considered under environmental factors, can impact developmental programming and result in later-life health outcomes [86,87]. Furthermore, prenatal and early life social conditions might lead to DNA methylation alterations involving immune functions and inflammatory pathways. Maternal depression in the prenatal period can affect DNA methylation of the brain-derived neurotrophic factor (BDNF) and maternal anxiety can induce loss of methylation in the *IGF2/H19* locus [71].

### 4.1. Germ-Line Associated Diseases

Epimutations within DMRs of imprinting genes are responsible for up to 50% of imprinting diseases (IDs), leading to alteration in the imprinted gene dosage. Defects in DNA methylation, as hyper- or hypomethylation, mainly localize within specific DMRs of the imprinted gene, and epigenetic mutations at further DMRs can account for the severity and outcome of the pathology [88]. Examples of IDs are Silver-Russell syndrome, displaying hypomethylation of the paternally imprinted locus *H19/IGF2* in the 11p15 region, together with other alterations at chromosome 7 and 11 [89,90,91,92], Fragile X syndrome, most commonly caused by a trinucleotide repeat expansion “CGG”, within the promoter of the fragile X mental retardation 1 protein (FMR1), which leads to aberrant DNA methylation and gene silencing [93,94,95], and Angelman syndrome, with a frequency of 2–3%, caused by an imprinting defect in the expression of maternally expressed 15q11-q13 genes. Additional examples of IDs are listed in Table 1.

### 4.2. Somatic Diseases

According to the “two-hits” model of cancer proposed by Knudson in 1971, a dominantly inherited germ-line mutation (first hit) and a second somatic mutation (second hit) predisposes and causes tumorigenesis, respectively [106]. However, evidence later proved that methylation-associated gene silencing can act as cancer-predisposing event (first hit) as effectively as genetic mutation [107,108,109]. Two distinct alterations of normal DNA methylation patterns occur in cancer: global hypo-methylation and gene-specific hypermethylation [110,111,112,113]. Genome-wide analyses evidenced that only 40 to 60% of a cancer cell genome is methylated, versus 80% of methylated genome in healthy controls. This global demethylation in cancer cells contributes to genomic instability, aneuploidy, an increased mutation rate, and could result from deregulation of passive and active demethylation processes mediated by DNMTs and TET family proteins, respectively [114,115,116]. Conversely, aberrant methylation of CGIs within 5′ regions of cancer-related genes is a hallmark of nearly all tumors and correlates with changes of chromatin structure that lead to silencing of tumor suppressor genes (TSGs) [117,118]. Colorectal cancer (CRC) and myelodysplastic syndrome (MDS) offer two good examples for understanding how aberrant loss or gain of DNA methylation may contribute to tumorigenesis, but they are not the only ones [119,120].

#### 4.2.1. Colorectal Cancer

CRC results from the accumulation of genetic and epigenetic alterations, both leading to genomic instability. Aberrant DNA methylation is considered a potential driver in CRC and understanding its establishment might contribute to developing new therapeutic strategies in the treatment of the tumor [121,122,123]. At the genome level, CRC is characterized by loss in DNA methylation (10–40% lower levels of absolute methylation than normal colonic tissue), mainly occurring within repetitive sequences and resulting in genomic instability and potential initiation of CRC [124,125]. In contrast, hypermethylation has been observed in regions corresponding to CGIs, histone H3 trimethylated on lysin 4 (H3K4me3), and open chromatin in normal controls [126]. A subgroup of CRCs, termed CpG-island methylator phenotype (CIMP), is characterized by aberrant promoter DNA methylation of critical genes involved in the WNT, P53, and RAS signaling pathways, although the role of component of these pathways, as initiators or drivers in the progression of the disease remains elusive [124,125].

#### 4.2.2. Myelodysplastic Syndrome

MDS is a clonal hematologic disorder often leading to acute myeloid leukemia (AML) in approximately 30% of cases. In contrast, with the common finding of global hypomethylation in cancer, MDS is characterized by a global hypermethylation associated with poor prognosis [127,128]. Aberrant promoter methylation of transcription factors, such as the CCAAT/enhancer-binding protein alpha (*CEBPA*), or TSG as *CDKN2B* (*P15*) and *CDKN2A* (*P16*), the adhesion molecule e-cadherin (*CDH1*) and the estrogen receptor (*ER*) [129] have been reported in either MDS and/or AML [129,130,131]. MDS displays extensive epigenetic reprogramming that explains why the number of hypermethylated genes is greater than in de novo AML or normal CD34+ cells. Hence, DNA methylation aberrations are linked to MDS progression, along with epigenetic regulators including *TET2*, *DNMT3a*, *IDH1*, *ASXL1,* and *EZH2* which are among the group of genes most frequently mutated in MDS [132,133]. In summary, genetic and epigenetic alterations often coexist, leading to distinct DNA methylation signatures in cancer cell genomes [134]. In terms of MDS treatment, the heterogenous nature of the disease warrants a complex combined therapy. Thus far, the DNMT inhibitors (DNMTi) 5-azacytidine (AZA) and decitabine (DAC), are the most successful epigenetic drugs approved for MDS and AML treatment. DNMTi modulate the epigenome of cancer cells by reversing aberrant DNA methylation patterns and re-establishing transcription of epigenetically silenced genes [135,136]. Additionally, a recent study performed in different AML cell lines has demonstrated that AZA is able to induce down-regulation of cell metabolism and up-regulation of immune defense-related genes. However, despite their efficacy, the severe side-effects of DNMTi, together with no or partial responses to treatment, do limit their clinical application [137].

## 5. Conclusions: The Best Is yet to Come

Epigenetics, defined as the interplay of DNA methylation, histone modifications, and expression of non-coding RNAs, govern numerous biological mechanisms from early development to old age, including biological response to endogenous or exogenous stimuli contributing to disease transition. In this review, we focused on DNA methylation, as alterations of this epigenetic mark are considered a triggering event in the pathogenesis of several human diseases affecting either germ-line imprinting disorders or somatic malignant transformation. The identification and characterization of different DNMT family members has led to a better understanding of mechanisms behind the establishment of DNA methylation in health and disease, and to the discovery of new therapeutic targets. DNMTs inhibitors have indeed been approved for the treatment of MDS and AML, while they are being tested in combination with other anticancer drugs, as a therapeutic approach for multiple solid cancers such as colon, ovarian, and lung cancer [138]. Unfortunately, the lack of specificity and the high cytotoxicity of the currently approved demethylating drugs hamper their clinical application.

The study of DNA methylation at single-cell level resolution is providing deeper insights into the epigenetic events driving disease onset and progression, and will likely shed light on other unknown features of DNA methylation. By means of new advanced technologies, such as WGBS and scBS, novel molecular targets will be identified and, more specific and less toxic therapeutic molecules might be developed with the ultimate goal of overcoming the downside effects of conventional hypomethylating protocols.

## Figures and Tables

**Table 1:** List of the best-known imprinting diseases and associated epigenetic lesions.

Imprinting Diseases	Epigenetic Lesions	Reference
Transient Neonatal Diabetes Mellitus Type 1 (TNDM1)	Hypomethylation of the maternally imprinted genes *PLAGL1* and *HYMAI*	[96,97]
Silver-Russell Syndrome	Hypomethylation of the paternally imprinted locus *H19/IGF2* and promoter hypomethylation of *HOXA4*	[91,92]
Beckwith-Wiedemann	Imprinting defects within two imprinted domains, *IGF2/H19* and *CDKN1C/KCNQ1OT1*	[98,99]
Fragile X Syndrome	De novo methylation of the *FMR1* gene	[93,95]
Angelman Syndrome	Imprinting defects within chromosome 15q11-q13 that alter the expression of the maternally inherited *UBE3A*	[100,101]
Prader-Willi Syndrome	Loss of expression of the paternally inherited chromosome 15q11.2-q13 due to imprinting defects	[102,103]
Pseudohypoparathyroidism	Epigenetic defects in the imprinted *GNAS* cluster on chromosome 20q13.3	[104,105]
